# Difficulty in Diagnosing and Treating a Prostate Abscess With Bacterial and Fungal Coinfection in an Immunocompromised Patient

**DOI:** 10.7759/cureus.21774

**Published:** 2022-01-31

**Authors:** Ryuichi Ohta, Shuzo Hattori, Keita Inoue, Chiaki Sano

**Affiliations:** 1 Communiy Care, Unnan City Hospital, Unnan, JPN; 2 Internal Medicine, Unnan City Hospital, Unnan, JPN; 3 Urology, Unnan City Hospital, Unnan, JPN; 4 Community Medicine Management, Shimane University Faculty of Medicine, Izumo, JPN

**Keywords:** renal insufficiency, prostate, immunocompromised state, contrast-enhanced computed tomography, coinfection, abscess

## Abstract

Prostate abscesses often occur in immunocompromised individuals. Contrast-enhanced imaging tests can aid diagnosis; however, they can be difficult to perform in older patients with renal insufficiency. Various organisms can cause prostate abscesses, and poor antibiotic penetration into the prostate can hinder treatment. Here, we report a case of prostate abscess manifesting as fever of unknown origin. The patient, a 78-year-old man with a history of heart failure, renal failure, and liver cirrhosis, presented with dyspnea and fever. He was initially diagnosed with aspiration pneumonia. However, the fever persisted, and urinary tract infection was diagnosed and treated with antibiotics and antifungal drugs. Further investigation with contrast-enhanced computed tomography revealed a prostate abscess. This case demonstrates the importance of aggressive investigation of fever of unknown origin in older patients with renal insufficiency. Furthermore, the problem of tissue penetration of antimicrobial agents should be thoroughly considered when treating prostate abscesses.

## Introduction

Prostate abscesses predominantly occur in immunocompromised individuals. Prostate abscesses can develop through direct invasion of organisms from the urinary tract or bacteremic dissemination [1]. The most common causative organisms are *Escherichia coli* and *Klebsiella pneumoniae*, which usually cause urinary tract infection (UTI) [1]. Men do not frequently experience UTIs; however, the presence of a UTI prompts further investigation because it is generally complicated in men. Moreover, in immunocompromised individuals, organisms can colonize the urinary bladder, causing UTI and prostate abscess [2]. Therefore, prostate abscesses should be considered a cause of symptoms of infection in immunocompromised men.

Contrast-enhanced imaging tests can aid the diagnosis of prostate abscess; however, they can be difficult to perform in older patients with renal insufficiency. Contrast-enhanced computed tomography (CT) is essential for diagnosing abscesses in deep parts of the body [3]. However, the contrast agent can cause contrast-induced nephropathy, especially in older patients [4], which may cause clinicians to avoid using contrast-enhanced imaging tests [5]. In addition, older patients tend not to experience the characteristic symptoms of prostate abscess [6]. Therefore, contrast-enhanced CT may not be performed in older patients with renal insufficiency who have a fever but no life-threatening symptoms.

Various organisms can cause prostate abscesses, and antibiotic penetration into the prostate can be an issue during treatment. The normal pH of the prostate is approximately 6.5, and in patients with prostatitis, it can increase to more than 8 because of chronic inflammation [7]. A high pH can inhibit antibiotic penetration into organs [8]. Therefore, prostate abscesses should be accurately diagnosed and treated with specific antibiotics. The prostate of immunocompromised patients can be infected by both bacteria and fungi, and it is difficult for low doses of antifungal drugs to penetrate the prostate [7,8]. Here, we discuss the case of an older immunocompromised patient who presented with a fever of unknown origin and was finally diagnosed with prostate abscess with bacterial and fungal coinfection.

## Case presentation

A 78-year-old man was admitted to our rural hospital because of a one-day history of fever (body temperature: 39.4°C), generalized pain, and rhinorrhea. On the day of admission, his fever persisted and pain increased, which affected his daily life. The patient independently performed his activities of daily living. He had a history of chronic heart failure, chronic renal failure due to hypertension, and chronic liver failure due to alcohol consumption. He was receiving furosemide (20 mg), spironolactone (50 mg), amlodipine (5 mg), and enalapril (5 mg).

On admission, the patient’s body temperature was 39.2°C, blood pressure was 110/72 mmHg, pulse rate was 78 beats per minute, respiratory rate was 16 breaths per minute, and peripheral oxygen saturation was 92%. Physical examination revealed pan-inspiratory crackles in the left lung field, white nails, and no edema of the extremities. Laboratory test results revealed a high C-reactive protein (CRP) level (14.24 mg/dL) and pyuria (Table 1).

**Table 1:** Laboratory parameters on admission. SARS-CoV-2: severe acute respiratory syndrome coronavirus 2

Marker	Level	Reference
White blood cells (×10^3^/μL)	5.60	3.5–9.1
Neutrophils (%)	79.5	44.0–72.0
Lymphocytes (%)	15.7	18.0–59.0
Monocytes (%)	4.6	0.0–12.0
Eosinophils (%)	0.0	0.0–10.0
Basophils (%)	0.2	0.0–3.0
Red blood cells (×10^6^/μL)	2.76	3.76–5.50
Hemoglobin (g/dL)	9.4	11.3–15.2
Hematocrit (%)	29.1	33.4–44.9
Mean corpuscular volume (fl)	105.4	79.0–100.0
Platelets (×10^4^/μL)	4.5	13.0–36.9
Total bilirubin (mg/dL)	0.5	0.2–1.2
Aspartate aminotransferase (IU/L)	34	8–38
Alanine aminotransferase (IU/L)	14	4–43
Lactate dehydrogenase (U/L)	275	121–245
Blood urea nitrogen (mg/dL)	38.6	8–20
Creatinine (mg/dL)	2.22	0.40–1.10
Estimated glomerular filtration rate (mL/min/L)	23.1	>60.0
Serum Na (mEq/L)	135	135–150
Serum K (mEq/L)	3.9	3.5–5.3
Serum Cl (mEq/L)	103	98–110
C-reactive protein (mg/dL)	14.24	<0.30
SARS-CoV-2 antigen	Negative	
Urine testing		
Leukocyte	3+	-
Nitrite	-	-
Protein (mg/dL)	300	-
Glucose	4+	-
Urobilinogen	-	-
Bilirubin	-	-
Ketone	-	-
Blood	3+	-
pH	5.5	
Specific gravity	1.031	

**Figure 1:**
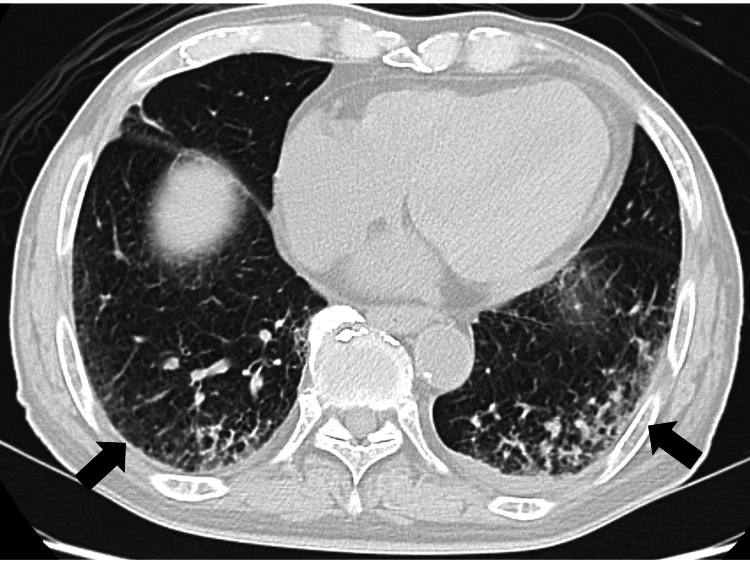
Chest computed tomography image showing infiltration in the bilateral lower lobes of the lungs.

Gram staining of a sputum sample revealed multiple white blood cells and a polymicrobial pattern. In contrast, gram staining of a urine sample revealed multiple white blood cells and gram-negative rods. Chest CT revealed bilateral infiltration in the lower lobes of the lungs (Figure 1) with no fluid retention in or around the prostate.

The patient was initially diagnosed with aspiration pneumonia and UTI and treated with piperacillin (PIPC; 4 g) and tazobactam (TAZ; 0.5 g) four times per day. Meanwhile, he did not need any urinary catheter.

After treatment, the patient’s fever decreased; however, his fatigue persisted. Sputum culture revealed no organism other than oral commensal bacteria, and urine culture revealed PIPC/TAZ-sensitive *Klebsiella pneumoniae*. Treatment with PIPC/TAZ was continued for 14 days. Subsequently, the patient’s body temperature increased above 39°C, but his fatigue persisted. Additionally, he needed rehabilitation for his fragility. On day 17 of hospitalization, Gram staining of a urine sample revealed fungi and white blood cells. Rectal examination revealed no tenderness of the prostate. The patient was diagnosed with UTI caused by *Candida* and treated with fluconazole (200 mg/day).

**Figure 2:**
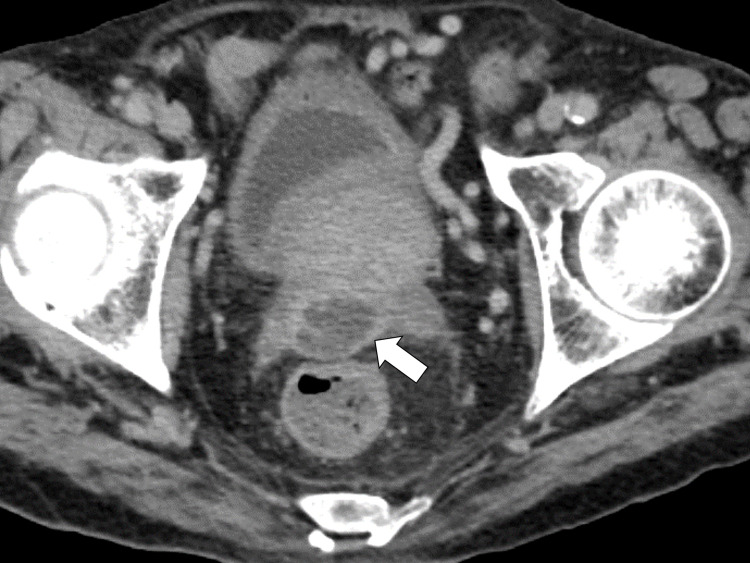
Pelvic computed tomography image showing fluid retention in the posterior part of the prostate.

Treatment with fluconazole was partially effective; the patient’s body temperature was maintained at approximately 37°C. Rectal examination revealed no tenderness of the prostate. Laboratory test results showed a CRP level elevation to 15.06 mg/dL, erythrocyte sedimentation rate of 152 mm/hour, and a creatinine level of 2.7 mg/dL. Although the patient’s renal function was severely impaired, we performed contrast-enhanced CT, which revealed fluid retention in the posterior part of the prostate (Figure 2).

**Figure 3:**
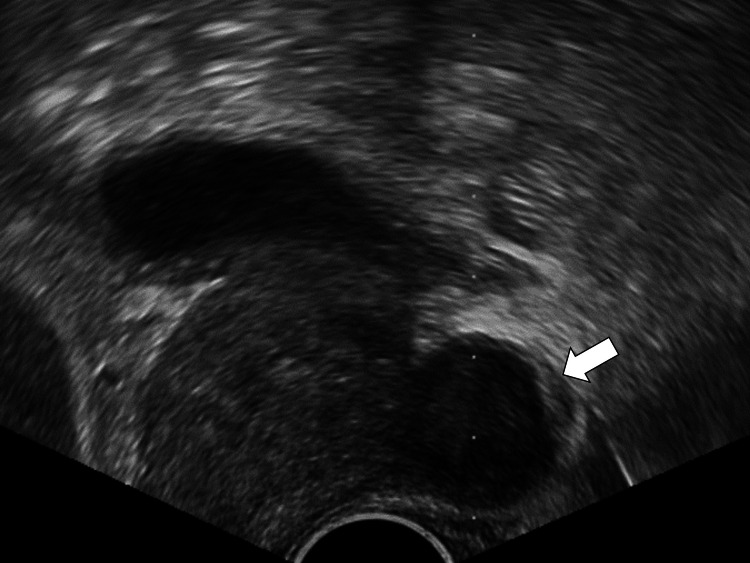
Image of ultrasound-guided fluid aspiration from the prostate.

We suspected that our patient had a prostate abscess and consulted a urologist. Ultrasound-guided aspiration (Figure 3) of green pus was performed.

**Figure 4:**
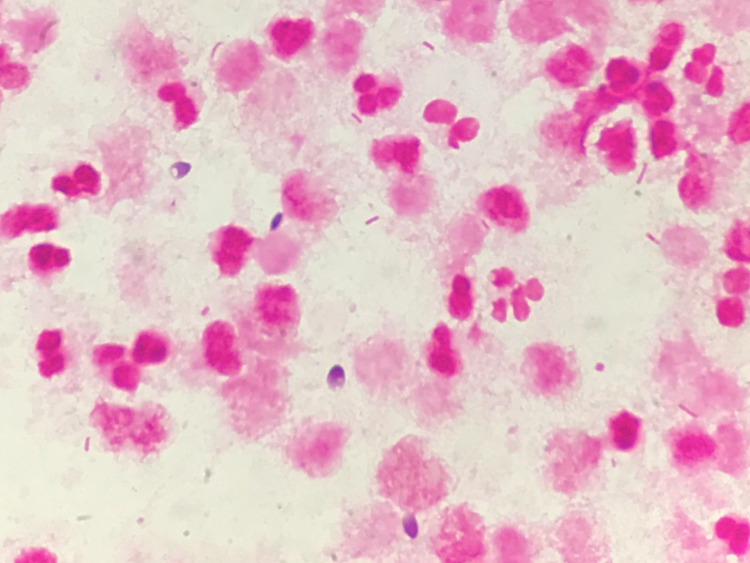
Gram-stained smear of pus aspirated from the prostate abscess showing white blood cells, gram-negative rods, and fungi.

Gram staining of the pus revealed white blood cells, gram-negative rods, and fungi (Figure 4).

We diagnosed the patient with prostate abscess with bacterial and fungal coinfection and treated him with levofloxacin (500 mg) and fluconazole (200 mg/day). Pus culture revealed *Klebsiella pneumoniae*, *Candida albicans*, and *Candida tropicalis*. The patient’s fever decreased; however, two days later, he developed chest pain and was eventually diagnosed with myocardial infarction (troponin I level: 48.9 ng/mL). The patient’s condition suddenly deteriorated and he died.

## Discussion

This case demonstrates the importance of aggressive investigation of fever of unknown origin in older patients with renal insufficiency. Furthermore, the problem of tissue penetration of antibiotics and antifungal drugs should be thoroughly considered when treating prostate abscesses.

Aggressive investigation and prompt appropriate treatment of fever of unknown origin are important in older patients with renal insufficiency. Because older patients do not exhibit typical symptoms, misdiagnosis occurs more often in them than in younger patients [6]. Additionally, older patients infrequently seek medical care and often attempt to independently control their symptoms [9-11]. As observed in this case, an immunocompromised state can mask various symptoms of life-threatening diseases, such as sepsis and abscess formation in the deep parts of the body [12]. Patients with renal failure who do not exhibit typical symptoms may not undergo intensive investigation, such as contrast-enhanced imaging, due to clinicians’ hesitation to perform these tests [13].

It is important to overcome the fear of exacerbating renal failure in older patients during the diagnosis of critical diseases. Contrast agent-induced renal damage can be irreversible and life-threatening in older patients with renal failure. However, the prevalence of contrast agent-induced irreversible renal damage may not be high; even including subclinical damage, it is unlikely to exceed 13% [14]. The risk of fever exacerbating critical diseases can be higher than the risk of renal damage [13]. Therefore, even in older patients with renal failure, contrast-enhanced imaging tests should be performed to diagnose critical diseases.

Various antibiotics and antifungal drugs cannot reach the prostate effectively; this problem should be thoroughly considered when treating prostate abscesses. Antibiotics do not penetrate well into the prostate [7]. Furthermore, the penetration of antifungal drugs into the prostate may be lower than that of antibiotics [7]. Abscess formation can further decrease the rate of penetration of antibiotics into the prostate [15]. In this case, PIPC/TAZ and fluconazole were used. In vitro studies revealed low penetration rates for these drugs. The rate of penetration of 13.5 g/day of PIPC/TAZ into the prostate is 50%; however, there are no data on patients with renal failure [16]. The rate of penetration of fluconazole into the prostate is approximately 3%; therefore, it can be challenging to treat fungal infections of the prostate through medical management alone [7]. These issues should be considered when treating patients with prostate abscesses.

When treating prostate abscesses, drug penetration and the patient’s immunological state should be considered. In this case, antifungal drugs and antibiotics were administered; however, because of low penetration into the prostate, the patient’s condition worsened. The patient’s immunocompromised state could have affected the pathophysiology [12]. In addition to a history of liver cirrhosis and renal failure, the patient had white nails, which is a predictor of mortality in admitted patients [17,18]. Therefore, the patient’s immunocompromised state might have facilitated the dissemination of *Candida *into the prostate [19]. As the penetration of antibiotics into the prostate can be low, the abscess should be quickly aspirated, and sufficient antibiotics and antifungal drugs should be promptly administered after diagnosis using contrast-enhanced imaging tests [20].

## Conclusions

This case demonstrates the importance of aggressive investigation of fever of unknown origin in older patients with renal insufficiency and the difficulty of treating prostate abscesses with bacterial and fungal coinfection. Although prostatic abscesses are rare, they should be considered during differential diagnosis in immunocompromised individuals. Therefore, drug penetration into the tissue should be considered when determining the dose of medication for treating a prostate abscess.
